# Electrophysiological Responses to Different Follicle-Stimulating Hormone Isoforms on Human Cumulus Oophorus Cells: Preliminary Results

**DOI:** 10.1055/s-0038-1676037

**Published:** 2018-12

**Authors:** Laura Silveira Ayres, Adriana Bos-Mikich, Nilo Frantz, Letícia Schmidt Arruda, Eloísa da Silveira Loss

**Affiliations:** 1 Department of Physiology, Instituto de Ciências Básicas da Saúde (ICBS), Universidade Federal do Rio Grande do Sul, Porto Alegre, RS, Brazil; 2 Embriology Laboratory, Nilo Frantz Research and Human Reproduction Center, Porto Alegre, RS, Brazil

**Keywords:** ovarian stimulation, endocrinology, ovarian follicles, cumulus cells, FSH, estimulação ovariana, endocrinologia, folículos ovarianos, células do cumulus, FSH

## Abstract

**Objective** The aim of the present study was to provide a better understanding of the specific action of two follicle-stimulating hormone (FSH) isoforms (β-follitropin and sheep FSH) on the membrane potential of human cumulus cells.

**Methods** Electrophysiological data were associated with the characteristics of the patient, such as age and cause of infertility. The membrane potential of cumulus cells was recorded with borosilicate microelectrodes filled with KCl (3 M) with tip resistance of 15 to 25 MΩ. Sheep FSH and β-follitropin were topically administered onto the cells after stabilization of the resting potential for at least 5 minutes.

**Results** In cumulus cells, the mean resting membrane potential was - 34.02 ± 2.04 mV (*n* = 14). The mean membrane resistance was 16.5 ± 1.8 MΩ (*n* = 14). Sheep FSH (4 mUI/mL) and β-follitropin (4 mUI/mL) produced depolarization in the membrane potential 180 and 120 seconds after the administration of the hormone, respectively.

**Conclusion** Both FSH isoforms induced similar depolarization patterns, but β-follitropin presented a faster response. A better understanding of the differences of the effects of FSH isoforms on cell membrane potential shall contribute to improve the use of gonadotrophins in fertility treatments.

## Abstract (pt)

**Resumo:**

**Objetivo** O objetivo do presente estudo foi fornecer uma melhor compreensão da ação específica de duas isoformas de hormônio folículo estimulante (FSH, sigla em inglês) (β-folitropina e FSH ovino) no potencial de membrana de células do cumulus oophorus humanas.

**Métodos** Dados eletrofisiológicos foram associados às características da paciente, como idade e causa da infertilidade. O potencial de membrana das células do cumulus foi registrado com microeletrodos de borossilicato preenchidos com KCl (3 M) com uma resistência de 15 a 25 MΩ. O FSH ovino e a β-folitropina foram administrados topicamente nas células após a estabilização do potencial de repouso durante pelo menos 5 minutos.

**Resultados** Nas células do cumulus, o potencial médio de membrana em repouso foi de -34,02 ± 2,04 mV (*n* = 14). A resistência média da membrana foi de 16,5 ± 1,8 MΩ (*n* = 14). O FSH ovino (4 mUI/mL) e a β-folitropina (4 mUI/mL) produziram despolarização no potencial de membrana 180 e 120 segundos após a aplicação do hormônio, respectivamente.

**Conclusão** Ambas as isoformas de FSH induzem padrões de despolarização semelhantes, mas a β-folitropina apresentou uma resposta mais rápida. Uma melhor compreensão das diferenças dos efeitos das isoformas do FSH no potencial da membrana celular contribuirá para aprimorar o uso das gonadotrofinas no estímulo ovariano controlado e em protocolos de maturação oocitária in vitro.

## Introduction

The preovulatory follicle is surrounded by several granulosa cell layers. A specific type of granulosa cells, cumulus oophorus, are firmly attached to each other and to the oocyte, surrounding it.^1^ The highly specialized cumulus cells have transzonal cytoplasmic projections (TZPs).^2^ These projections cross the zona pellucida and reach the oolemma. The TZPs present gap junctions at their endings, which allow the transfer of low-molecular weight molecules between the oocyte and the cumulus cells.^2^ The communication between the cumulus cells and the oocyte is essential for the development of the follicle, for the maturation process of the oocyte, and for fertility.^1^ Otherwise, the complete maturation of the follicle only occurs in the presence of follicle stimulating hormone (FSH).^1^ In females, FSH has only one well known target: the follicle granulosa cells (which include cumulus cells), in which the gonadotrophin initiates and mediates multiple functions required for the maturation of the oocyte.^1^

Before it is released into the circulation, the FSH molecule is glycosylated by the addition of oligosaccharides in two N-linked glycosylation sites in each FSH subunit.^3^ Each carbohydrate branch added to the molecule may end in a negatively charged sialic acid residue, conferring different isoforms of the FSH, with different isoelectric points.^3^ It is already known that the effect of FSH on in vitro follicle culture depends on the degree of purity of the commercial preparations.^4^ Besides, not only the hormone concentration, but also its quality, isoform type, and purity have different effects in the early phase of follicular development.^4^

Electrophysiological studies may provide an additional understanding of the mechanism of hormonal action. The action of FSH on the granulosa cells of swine was associated with a raise of intracellular Ca^2+^.^5^ Other studies using immature Sertoli cells from rats have shown that FSH causes depolarization in the membrane potential, which is associated with L-type voltage-gated Ca^2+^ channels (L-VDCC).^6^ ^7^ However, to date, no studies evaluating the action of FSH on ionic channels in human cumulus oophorus cells have been found. Based on these previous studies, the aim of the present study was to standardize the intracellular electrophysiological register technique to human cumulus cells and to evaluate the effects of two FSH isoforms (β-follitropin and sheep FSH) on the membrane potential of cumulus cells. Sheep FSH was previously tested in Sertoli cells^6^ ^7^ and presents a different isoelectric point from β-follitropin, which is used in human ovarian stimulation protocols. In addition, the electrophysiological data obtained in the present study were associated with some characteristics of the patient, such as age and cause of infertility.

## Methods

### Study Design

This is an experimental study.

### Setting

The cumulus oophorus cells were obtained from an assisted reproduction center (Nilo Frantz Research, Porto Alegre, RS, Brazil). The present study was approved by the ethics committee of the Universidade Federal do Rio Grande do Sul (UFRGS, in the Portuguese acronym), with the process number 20173.

### Participants

The criteria of eligibility for the present study were: consenting patients, assigned to intracytoplasmic sperm injection (ICSI). All of the patients who participated in the study signed the informed consent form, approved by the Ethics Committee of the UFRGS (process number 20173), before the beginning of the procedures.

### Variables

A data bank containing information on age, cause of infertility, number of mature oocytes (MII), number of normal fertilized oocytes (2-pronuclei), number of embryos graded from 1 to 5, obtained from the medical history of the patients, and mean membrane potential of cumulus cells at rest was organized.

### Study Size

For the intracellular registration experiments, the treatments were repeated at least 4 times (*n* = 4). The sample calculation was performed with WINPEPI software version 9 (Abramson JH and Peritz E, Hebrew University and Hadassah faculty of Medicine, Jerusalem, Israel), using a sample power of 80% and a confidence interval (CI) of 95%.

#### Cumulus Oophorus Cells

**Fig. 1:**
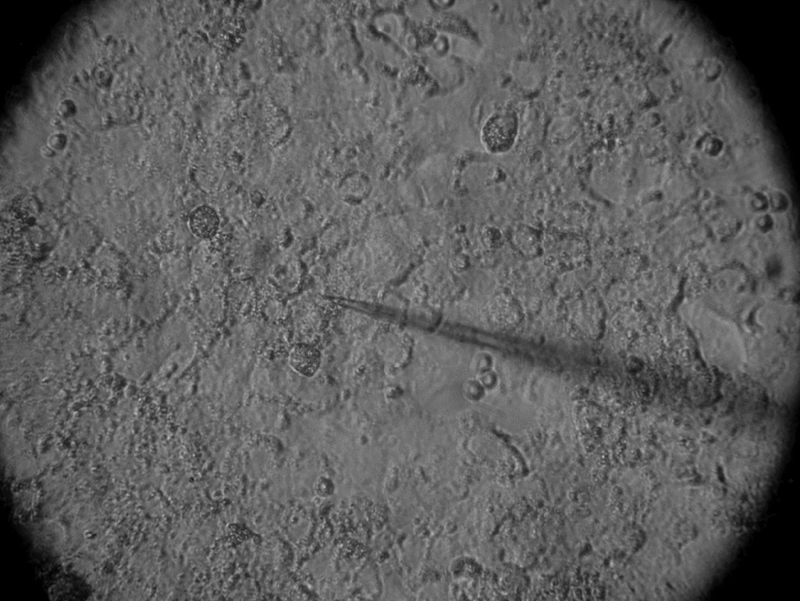
Cumulus cells attached to the bottom of a culture dish for electrophysiological recordings.

The collection of the oocytes was performed between 10 and 14 days after ovarian stimulation. Pituitary suppression was achieved using a gonadotropin-releasing hormone (GnRH) antagonist, and ovarian stimulation was achieved using recombinant FSH (rFSH). When at least one follicle reached 18 mm in diameter, the patients received a single dose of human chorionic gonadotropin (hCG) (10,000 IU). The collection of the oocyte was performed 36 hours after the administration of hCG, and the insemination was performed by ICSI. After denudation, the cumulus cells were placed in a culture dish in human tubal fluid (HTF) medium (Life Global, Guilford, CT, USA) with 10% synthetic serum substitute (SSS) (Life Global, Guilford, CT USA) and left to attach to the bottom of the dish for between 24 and 48 hours, as shown in Fig. 1.

### Solutions and Hormones

Sheep FSH (50 UI) (Sigma, St. Louis, MO, USA), and β-follitropin (625 UI/mL) (Puregon, Merck/Schering-Plough, North Wales, PA, USA) were used at a final concentration of 4 mUI/mL. Hank’s Balanced Salt Solution (HBSS) contained: CaCl_2_ · 2H_2_O, MgSO_4_ (anhyd), KCl, KH_2_PO_4_ (anhyd), NaHCO_3_, NaCl, Na_2_HPO_4_ (anhydrous), D-Glucose and Phenol Red · Na (H9269-1L, Sigma, St. Louis, MO, USA). Sodium hydroxide (NaOH [1N]) was added to this solution to reach a pH of 7.4.

### Electrophysiological Experiments

The dish containing the cumulus cells was positioned in a Nikon Diaphot-TMD inverted microscope (Nikon Corporation, Tokyo, Japan) and connected to a perfusion pump tubing. The dish was then perfused with 1 mL/min of HBSS with HEPES and maintained at 37° C in water bath (DeLeo & Cia Ltda., Porto Alegre, RS Brazil). Borosilicate microelectrodes were filled with KCl (3 M) with a tip resistance of 15 to 25 MΩ. The intracellular recording of each cell was amplified using an Intra 767 WPI intracellular amplifier (World Precision Instruments Inc., Sarasota, FL, USA). Square current pulses of 0.5 nA, 0.5 Hz, and 250 milliseconds were applied by the microelectrode to estimate membrane resistance using the S48 stimulator (Grass Instrument, West Warwick, RI, USA). A Tektronix TDS 210 2-Channel Digital Oscilloscope (Tektronix, Beaverton, OR, USA) and the Wavestar Lite software, Version 1.0.10 (Tektronix, Beaverton, OR, USA) were used to record the variations in the membrane potential. Sheep FSH and β-follitropin were topically administered onto the cells after the resting potential was stabilized for at least 5 minutes. Each treatment was repeated at least four times with different cells from different patients, and the variations in the membrane potential were recorded. Each cell was tested with one FSH isoform. The results are presented as mean ± standard error of the mean (SEM).

### Statistical Analysis

Statistical analyses were performed by one-way analysis of variance (ANOVA) with the Bonferroni posttest or with the Fischer exact test. The analyses were performed using GraphPad InStat version 3.01, 32 bits for Windows 95/NT (GraphPad Software, San Diego, CA, USA). Differences were considered significant if *p* < 0.05.

## Results

### Participants

**Fig. 2:**
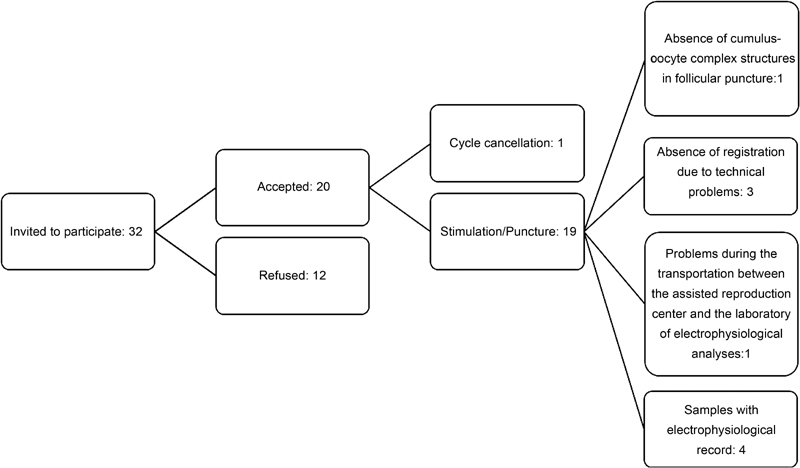
Flowchart of patient selection and electrophysiological data.

The resting membrane potential of the cumulus cells from 14 patients was recorded (Fig. 2 presents a flowchart of patient selection and electrophysiological data). Of these patients, six presented a male cause of infertility and eight presented female infertility. The age of the patients, the number of oocytes collected, the number of mature and fertilized oocytes, as well as the number of embryos grades 1 and 2 or grades 3, 4, and 5, human chorionic gonadotrophin (hCG) test results, and the mean cellular membrane potential recorded are shown in Table 1.

**Table 1:** Descriptive data and follow-up of patients and samples

Patient	Age (years old)	Infertility cause	Number of oocytes	MII	2PN	Embryo grades 1 and 2	Embryo grades 3, 4 and 5	hCG mlU/ml	Mean membrane potential (mV)
1	28	PCOS	33	21	17	3	15	No ET	−35.83
2	39	OI	7	7	7	3	4	No ET	−54.63
3	37	MF	15	12	7	2	7	< 5	−15.3
4	42	MF + OI	8	8	6	0	2	113	−23.22
5	31	CR	21	8	0	0	0	No ET	−14.5
6	39	PCOS	19	18	12	5	7	No ET	−17.71
7	25	MF	7	6	5	4	1	No ET	−15.02
8	39	PCOS	12	9	8	5	3	No ET	−14.08
9	41	E	6	4	2	1	1	No ET	−17.84
10	31	TF	6	5	5	1	4	No ET	−11.97
11	34	MF	8	7	6	1	5	347,74	−17.04
12	32	TF + UN	7	6	2	1	2	150	−8.95
13	30	MF + PCOS	8	8	3	2	0	99	−36.29
14	35	MF	21	17	14	10	6	No ET	−6.65

Abbreviations: 2PN, fertilized oocytes (presenting 2-pronuclei); CR, cryopreservation; E, endometriosis; ET, embryo transfer; hCG, human chorionic gonadotrophin results; MF, male factor; MII, metaphase II oocytes; OI, ovarian insufficiency; PCOS, polycystic ovary syndrome; TF, tubal factor; UN, unexplained.

### Clinical Variables

The analysis of the resting membrane potential of the cumulus cells revealed one group of patients presenting less negative membrane potential (-6 to -16 mV), and the other group presenting a more negative membrane potential (-16 to -60 mV). A comparison between some of the characteristics of the patients from the two groups was made. It was observed that in patients with male infertility factor, most of the cells have less negative membrane potentials, whereas in the cases of polycystic ovary syndrome (POS), most of the cells have more negative membrane potentials (Table 2). Comparing the membrane potential with the age of the patients, a slight difference was observed. Women aged between 20 and 35 years old showed a tendency to present cells with less negative membrane potential when compared with older women (Table 3). The comparison between the number of immature and mature oocytes (MII) from patients with male factor of infertility and from patients with POS presented no significant difference (*p* = 0.0941, odds ratio [OR] =1.792; 95%: CI: 0.9110–3.525). There was also no difference in oocyte maturity status between patients with male factor of infertility and with female factor of infertility (except POS) (*p* = 0.1018; OR = 2.133; 95% CI: 0.8962–5.078). In addition, the number of fertilized oocytes (2-pronuclei) and unfertilized oocytes from patients with male factor of infertility and with POS (*p* = 0.6914; OR = 0.6250; 95% CI: 0.1075–3.634) or with female factor of infertility (except POS) (Fischer exact test: *p* = 1.0000; OR = 1.375; 95% CI: 0.1133–16.121) presented no significant difference. For the number of embryos with better viability (grades 1 and 2) and less viability (grades 3, 4 and 5), there was also no statistical difference between the male factor, the POS or the non-POS groups (*p* = 0.4866, OR = 1.491; 95% CI: 0.5969–3.725).

**Table 2:** Comparison between membrane potential and infertility factors

Membrane potential (mV)	Male Factor	Female PCOS	Factor Non-PCOS	Total
−6.0 to −16.0	4 (40%)	1 (11%)	2 (20%)	7 (50%)
−16.1 to −60.0	1 (10%)	3 (33%)	3 (30%)	7 (50%)
Total	5 (50%)	4 (44%)	5 (50%)	14 (100%)

Abbreviation: PCOS, polycystic ovary syndrome.

**Table 3:** Comparison between membrane potential and patient age range

Membrane potential (mV)	20–35 years old	> 35–40 years old	Total
−6.0 to −16.0	5 (36%)	2 (14%)	7 (50%)
−16.1 to −60.0	3 (21%)	4 (29%)	7 (50%)
Total	8 (57%)	6 (43%)	14 (100%)

### Basal Electrophysiological Values of the Membrane of Human Cumulus Cells 

**Fig. 3:**
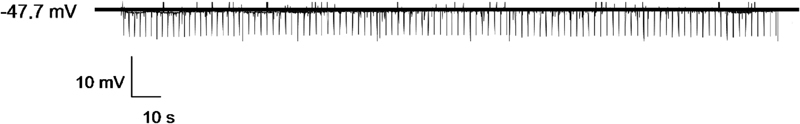
Recording of the membrane resting potential of a typical cumulus cell with −47.7 mV. The vertical lines provide the membrane input resistance values achieved by the application of pulses of 0.5 nA.

In our experimental conditions, the basal electrical characteristics of the membrane of the cumulus cells were: resting membrane potential of −34.02 ± 2.04 mV (*n* = 14); and resting membrane resistance of 16.5 ± 4.03 MΩ (*n* = 14). These values remained steady for at least 5 minutes before the administration of the hormone (Fig. 3).

### Effect of Sheep FSH in the Membrane Potential of Human Cumulus Cells

**Fig. 4:**
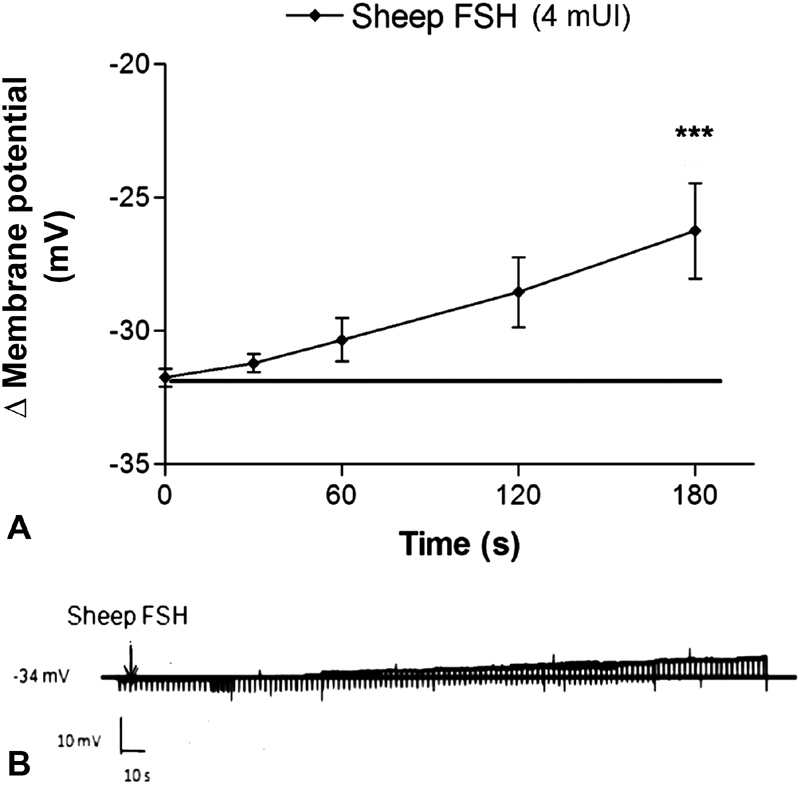
Effect of sheep FSH on the membrane potential of cumulus cells. (**A**) Depolarizing effect of sheep FSH at 4 mUI/mL on the membrane potential of cumulus cells compared with the resting potential (*** *p* < 0.001) (*n* = 5). (**B**) Recording of typical cumulus cell membrane potential during the administration of sheep FSH (4 mUI/mL).

Sheep FSH (4 mUI/mL) induced depolarization in the membrane potential of cumulus cells. This response was significantly different from the resting value described above, after 180 seconds of FSH administration (Fig. 4A and B). The resistance of the membrane of the cumulus cells was not significantly affected by the experimental conditions.

### Effect of β-follitropin in the Membrane Potential of the Cumulus Cells

**Fig. 5:**
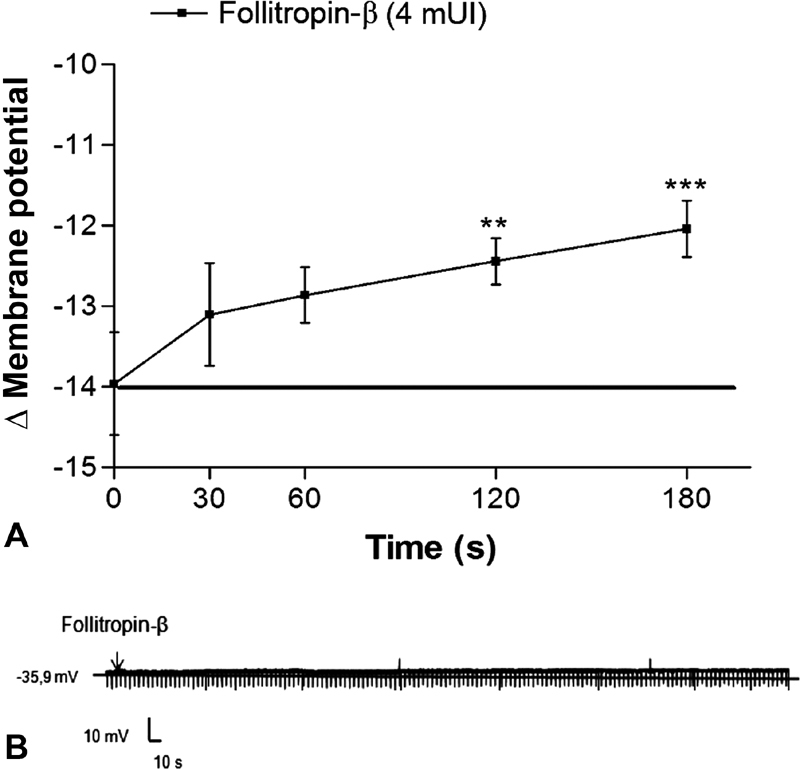
Effect of β-follitropin on the membrane potential of cumulus cells. (**A**) Depolarizing effect of β-follitropin at 1 µM on the membrane potential of cumulus cells compared with the resting potential (** *p* < 0.01; *** *p* < 0.001) (*n* = 4). (**B**) Recording of typical cumulus cell membrane potential during the administration of β-follitropin (4 mUI/mL).

Beta follitropin (4 mUI/mL) induced membrane depolarization in cumulus cells. This effect was significantly different from the resting value after 120 seconds of β-follitropin administration (Fig. 5A and B). The resistance of the membrane of the cumulus cells was not significantly different under the experimental conditions.

## Discussion

The standardization of the electrophysiological register technique for the cumulus cells was successfully achieved and the mean resting membrane potential obtained was −34.02 ± 2.04 mV (SEM). The mean resistance of the membrane to the ion flow was 16.5 ± 1.8 MΩ (SEM). Sheep FSH application (4 mUI/mL) led to a statistically significant slow depolarization 180 seconds after the administration of the hormone (*p* < 0.01). The administration of β-follitropin (4 mUI/mL) led to a statistically significant slow depolarization 120 and 180 seconds after the application of the hormone (*p* < 0.001). The depolarization pattern was similar between both isoforms. Beta follitropin had a more immediate effect than sheep FSH.

The limitations of the present study were the small sample size (further investigations using a large cohort are needed), the inclusion of participants with different clinical variables that may have interfered with the results (age, and cause of infertility), and that the data were obtained from samples collected after controlled ovarian stimulation, which may not necessarily be extrapolated to natural cycles.

The standardization of the intracellular electrophysiological register technique to human cumulus oophorus cells was achieved based on previous studies using immature Sertoli cells from rats.^6^ ^7^ The pretreatment of the culture dishes was not necessary, since the cells adhered to the bottom of the dish by themselves (Fig. 1). Gilula et al (1978)^8^ used rat cumulus-oocyte complexes pretreated culture dishes with poly(L-lysine). Their report is, to our best knowledge, the only previous study using the intracellular register technique in cumulus cells. However, there are several differences between their study and the present one. The most effective electrode tip resistance value was found to range between 15 and 25 MΩ, while Gilula et al (1978)^8^ used electrodes with resistances ranging between 50 and 70 MΩ. In human cumulus cells, the average membrane potential obtained was −34.02 ± 2.04 mV (Fig. 2). This resting membrane potential was different from that observed in rat cumulus-oocyte complexes, which was −50 to −60 mV.^8^ However, it has to be taken into account that the present experiments were performed using isolated human cumulus cells, while Gilula et al (1978)^8^ used rat cumulus-oocyte complex, observing an ionic coupling between cumulus cells and the oocyte. The average membrane resistance of human cumulus cells in the present study was 16.5 ± 1.8 MΩ.

The isoforms of FSH induced a rapid depolarizing effect on the membrane of human cumulus cells (Fig. 3 and Fig. 4). Even though the responses were similar to both isoforms, the action of β-follitropin was apparently faster than that of sheep FSH. The depolarizing effect of both FSH isoforms achieved their maximum at 180 seconds and returned to the resting potential at ∼ 300 seconds. The action of FSH on the membrane potential has previously been studied in Sertoli cells from immature rats.^6^ In these cells, FSH induces biphasic membrane potential changes. Very short hyperpolarization, with the duration of seconds, occurs followed by a prolonged depolarization (> 6 minutes).^6^ ^9^ The hyperpolarization was blocked by tolbutamide, an inhibitor of ATP-sensitive K^+^ channels (K^+^ATP).^7^ The FSH-induced depolarization in the membrane of Sertoli cells was nullified by verapamil, a voltage-dependent calcium channel blocker.^9^ Therefore, FSH-induced depolarization in immature Sertoli cells is related to the uptake of Ca^2+^ through voltage-dependent calcium channels.^6^ ^9^ The same mechanism may be involved in FSH-induced depolarization in cumulus cells, which may be evaluated in future studies.

Using swine granulosa cells, Flores et al (1990)^5^ found that FSH raises the intracellular calcium concentration, and that this effect was completely abolished by verapamil. The expression of a variety of Ca^2+^-sensitive K^+^ channels was observed in human granulosa cells.^10^ These channels are associated to the production of sex hormones, which is stimulated by gonadotrophins.^10^ In addition, another study observed the presence of K^+^ATP in human granulosa cells.^10^ All those previous studies assessed ionic channels without relating their findings with the different FSH isoforms, which was the main objective of the present study.

Comparing the depolarization induced by sheep FSH and the one induced by β-follitropin, one can observe a similarity in the pattern of depolarization between the two isoforms, whereas β-follitropin had a faster effect. Nevertheless, there was no statistical difference in the depolarization effect between the 2 isoforms at 120 and 180 seconds. Sheep FSH is a less purified mixture of FSH isoforms. On the other hand, β-follitropin is a recombinant human FSH (rhFSH) produced by a Chinese hamster ovary cell lineage transfected with two plasmids containing genes for α and β FSH chains. Beta follitropin is composed by two times less acidic isoforms and a proportion two times higher of less acidic isoforms than FSH from the urine of postmenopausal women (urofollitropin).^11^ This may explain the differences in the pattern of depolarization between the isoforms.

A previous study using rat and mouse ovarian follicles showed that naturally occurring FSH isoforms can have different, and even opposite effects in target cells.^12^ It was shown that less acidic isoforms (pH 6.6–4.6) were able to induce higher cyclic adenosine monophosphate (cAMP) release, higher estrogen production, and higher activity of citochrome P450 aromatase than more acidic isoforms (pH > 7.10). On the other hand, more acidic isoforms induced a higher expression of α-inhibin RNA messenger. Concerning in vivo effects, less acidic isoforms were as effective as or more effective than more acidic isoforms in sustaining rat granulosa cell proliferation when administered immediately after hypophysectomy.^12^ A higher activity of less acidic FSH isoforms, compared with more acidic isoforms, was also observed related to other parameters of hormonal actions.^13^ ^14^ ^15^ The present report also observed a tendency to an earlier effect of the less acidic FSH isoform (β-follitropin). Cruz et al analyzed the gene expression profile in cumulus cells according to the type of gonadotropin received during ovarian stimulation and revealed greater differences between the urinary FSH (uFSH) and the human menopausal gonadotropin (hMG) groups compared with the rest of the pairwise comparisons; rFSH versus hMG and uFSH versus rFSH.^16^ Their results suggest that controlled ovarian stimulation induces specific gene expression profiles in human cumulus cells depending on the type of gonadotropin used.^16^ The choice of different isoforms to modulate the activity of cumulus cells may be a useful tool for both in vivo and in vitro oocyte maturation. More studies on the FSH electrophysiology of human cumulus cells are necessary to clarify the different action mechanisms of FSH isoforms.

A tendency toward a higher percentage of cells with a more negative mean resting membrane potential was observed in patients with female factor of infertility, a result that might be further explored, with the inclusion of a greater number of patients and the evaluation of the differences in the membrane channels between fertile and subfertile patients. Also, older women (36–50 years old) seem to have a higher percentage of cells with more negative mean resting membrane potential than younger women (20–35 years old). A previous report that assessed the capacity of FSH to affect the expression and the internalization of gap junctions in hypophysectomized rat granulosa cells observed that FSH and luteinizing hormone (LH) may have antagonistic effects in gap junctions.^17^ The authors concluded that during the initial follicular growth, FSH stimulates the expression of gap junctions in the cell surface, while gap junction renewal occurs during the later stages of follicular growth.^17^ Older patients and those affected by ovarian illnesses generally have an increased FSH production to trigger and improve folliculogenesis through a greater ovarian stimulation.^18^ Similarly, cells obtained from older patients may present an altered expression of other molecules, as well as of ionic channels, leading to changes on the resting membrane potential.

Several studies using FSH isoforms demonstrated that the development of normal follicles and of healthy oocytes depends on the balanced distribution of isoforms in specific moments of the follicular maturation.^3^ ^19^ ^20^ ^21^ In addition, although uFSH isoforms have been used successfully for years, recombinant human FSH (r-hFSH) have presented better results and safer use.^22^ ^23^ On the other hand, another study, which included only women > 37 years old, observed that the patients treated with uFSH had significantly higher rates of 2PN zygotes, of grade І embryos, and of endometrial thickness on the day of hCG application, and a lower rate of no transferable embryos (1.2 versus 5.3%, *p* = 0.019) than women treated with recombinant follicle stimulating hormone (rFSH).^24^ In agreement with this study, Colacurci et al,^25^ in a study with women between 35 and 40 years old, performed a standard downregulation with a GnRH-analogue and assigned 115 women to stimulation with uFSH for 6 days and then shifting to rFSH (group A).^25^ Other 115 women underwent a stimulation protocol with only rFSH (group B).^25^ In this study, the number of days of stimulation was lower in group A than in group B, there was a higher proportion of MII oocytes and of grade 1 embryos, higher implantation and pregnancy rates in group A versus group B, concluding that a sequential protocol using uFSH in the early days of stimulation and, subsequently, rFSH, may improve the in vitro fertilization (IVF) outcome in patients of advanced reproductive age.^25^ In the present study, the tendency of a more negative resting membrane potential in older women is indicative of the possible different responses to FSH according to the age.

Wang et al^16^ compared the glycosylation of urinary human FSH (uhFSH), obtained from human urine with that of rhFSH. They showed that highly sialylated, branched, and macro-heterogeneity glycans are predominant in the uhFSH, compared with rhFSH, as well as a high degree of heterogeneity in the N-glycopeptides of both human FSH isoforms.^16^ The earlier depolarization of β-follitropin in this study indicates a difference in action between FSH isoforms. Future studies may further explore the specific responses to FSH isoforms according to age ranges and for which age and infertility causes each isoform is recommended.

In the present study, there were no differences in membrane potential, number of immature and mature oocytes, number of fertilized oocytes, and number of embryos with better viability (grades 1 and 2) between patients with male and female infertility causes. This may be due to the small sample size, but in terms of electrophysiology, it is a good aspect, indicating homogeneity between the patients evaluated.

Although the function of ionic currents in oocyte maturation is still unclear, the changes in the electrical characteristics of the plasma membrane seem to be involved in oocyte growth, in meiosis progression, and in the preparation for fertilization.^26^ A better knowledge of electrical properties during follicle growth may help to develop new culture systems for in vitro oocyte maturation protocols and improved ovarian stimulation regimens.^26^ It has also been demonstrated that FSH intersects with the follicular epidermal growth factor network to activate the phosphatidylinositol 3-phosphate/AKT cascade in the oocyte to control translation and developmental competence, providing a molecular rationale for the use of FSH to improve egg quality in vitro.^27^

## Conclusion

The above reports and results encourage us to continue the present research, to investigate the potential relationship between infertility factor, age and cumulus cell membrane potential registers, as well as the influence of different FSH isoforms on electrical signaling and, consequently, oocyte maturation.
